# Concomitance of Persistent Primitive Olfactory Artery and Basilar Artery Fenestration

**DOI:** 10.7759/cureus.94925

**Published:** 2025-10-19

**Authors:** George Triantafyllou, Nikolaos-Achilleas Arkoudis, George Tsakotos, Georgios Velonakis, Maria Piagkou

**Affiliations:** 1 Department of Anatomy, School of Medicine, Faculty of Health Sciences, National and Kapodistrian University of Athens, Athens, GRC; 2 Second Department of Radiology, School of Medicine, “Attikon” University General Hospital, National and Kapodistrian University of Athens, Athens, GRC

**Keywords:** basilar artery, cerebral arterial circle, fenestration, persistent primitive olfactory artery, variation

## Abstract

The cerebral arterial circle exhibits substantial morphological variability, occasionally encompassing rare congenital vascular variants. We report a unique case of a 64-year-old woman in whom computed tomography angiography (CTA) revealed the coexistence of a persistent primitive olfactory artery (PPOA) and basilar artery (BA) fenestration. The PPOA originated from the left anterior cerebral artery (ACA) and coursed anteroinferior toward the cribriform plate, where it formed a characteristic hairpin turn before resuming the typical A2 trajectory. Concurrently, a BA fenestration was identified, with the right anteroinferior cerebellar artery arising from the right limb of the fenestrated segment. The simultaneous occurrence of these two vascular morphological variants is a unique phenomenon. The recognition of such variations is critical, as both may alter cerebral hemodynamics, predispose to aneurysm formation, and complicate surgical or endovascular interventions. The awareness of these vascular variants is essential for accurate diagnosis and surgical planning. CTA and magnetic resonance angiography (MRA) remain reliable, non-invasive imaging modalities for the identification and characterization of these conditions.

## Introduction

The cerebral arterial circle (circle of Willis) is formed by the convergence of the internal carotid and vertebrobasilar systems, ensuring collateral circulation within the brain. The internal carotid artery (ICA) gives rise to the anterior cerebral arteries (ACAs) and middle cerebral arteries (MCAs), which constitute the anterior circulation. In contrast, the vertebral arteries (VAs) unite to form the basilar artery (BA). The BA subsequently bifurcates into the bilateral posterior cerebral arteries (PCAs), completing the posterior circulation. Moreover, the anterior communicating artery (AComA) interconnects the two ACAs, and the posterior communicating arteries (PComA) anastomose the ICA with the PCA [1].

This adult vascular configuration results from a complex embryological process involving multiple transient arterial networks that remodel and regress during development. Primitive ICA supplies the developing brain via two main branches: a rostral branch, which corresponds to the primitive olfactory artery, and a caudal branch, which contributes to the formation of the posterior circulation. The primitive olfactory artery supplies the prosencephalon during early embryogenesis and typically regresses as the definitive ACA develops [2,3].

In rare instances, failure of regression results in the persistent primitive olfactory artery (PPOA) into adulthood. This vascular anomaly has an estimated prevalence of 0.14%, as reported in a previous single-center retrospective angiographic study [3].

Although isolated cases of PPOA have been described, only a few reports have documented its coexistence with other cerebral arterial circle variants, such as accessory MCAs or azygos ACAs [2,3]. The present report adds to this limited body of literature by describing a unique case that was not previously documented.

## Case presentation

During a retrospective angiographic study based on a computed tomography angiography (CTA) archived lot, the scan of a 64-year-old female patient was anatomically investigated. The scans were documented using Horos software version 3.3.6 (Horos Project, Annapolis, MD). Evidence was gathered from the multiplanar reconstruction of the axial, coronal, and sagittal slices, as well as their three-dimensional volume reconstruction.

**Figure 1:**
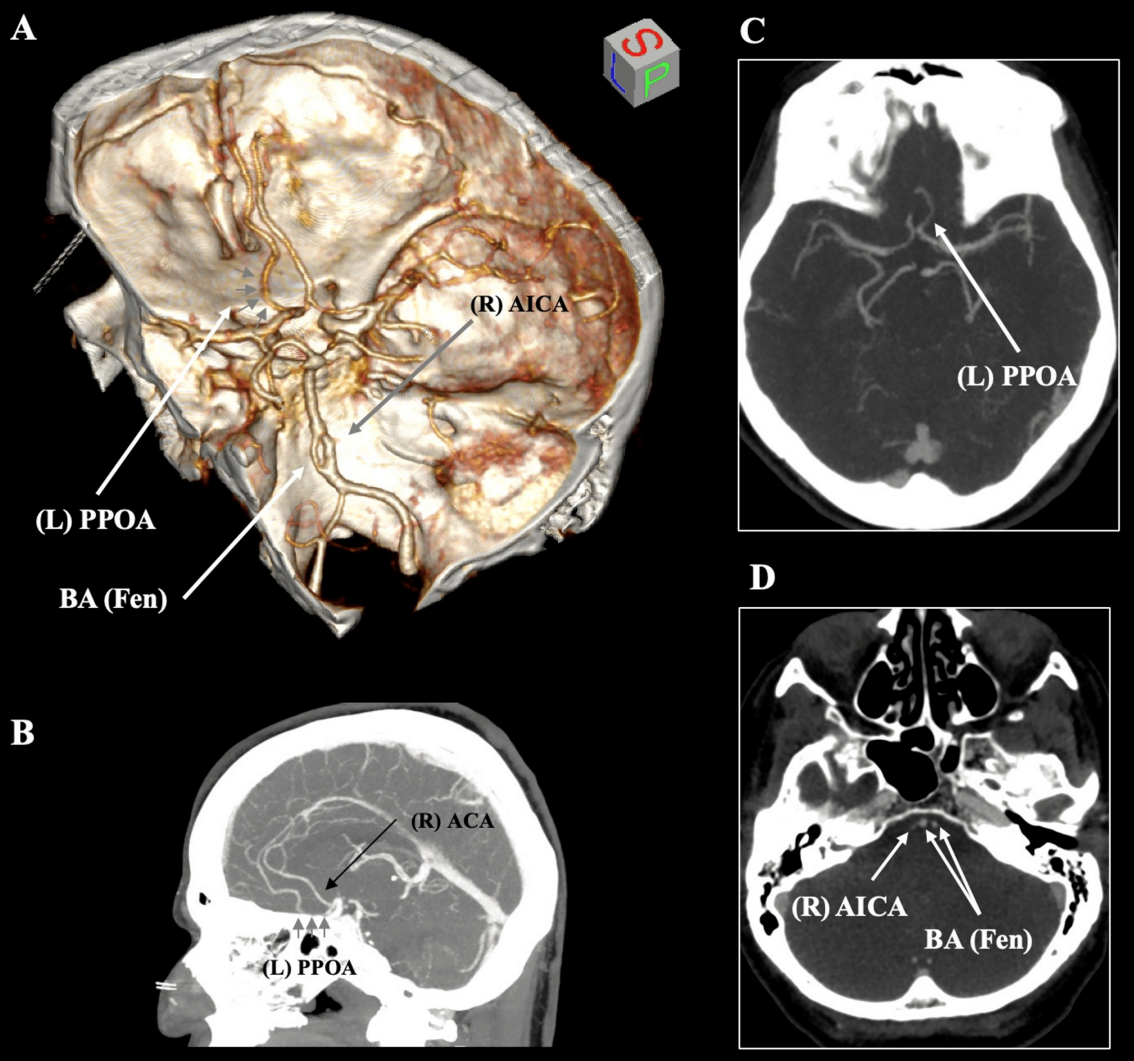
Computed tomography angiography of the 64-year-old female patient. (A) Three-dimensional volume rendering of computed tomography angiography (CTA) showing the left persistent primitive olfactory artery (PPOA, grey arrows) arising from the left anterior cerebral artery and coursing anteroinferiorly toward the cribriform plate. The basilar artery (BA) demonstrates a proximal fenestration (Fen). (B) Sagittal CTA reconstruction illustrates the left PPOA (grey arrows) with its characteristic hairpin turn and the typical course of the right anterior cerebral artery (ACA). (C) Maximum intensity projection of the axial slice presenting the PPOA. (D) Axial slice showing the BA fenestration with its two limbs. AICA, anteroinferior cerebellar artery; L, left; R, right

On the anterior circulation, the bilateral ACAs typically originate from the ICA system. The left-sided ACA had a diameter of 2.21 mm, and the right-sided ACA had a diameter of 1.77 mm (at their origin). The ACAs were typically anastomosed with the AComA. The left-sided A2 (post-communicating) segment had an abnormal course with an anteroinferior trajectory toward the cribriform plate of the ethmoid bone. Then, it had a hairpin turn to obtain its typical A2 course; thus, this course corresponded to a PPOA (Figure 1).

On the posterior circulation, the VAs typically unite to form the BA. Distally to its formation (4.94 mm), the BA was fenestrated within a length of 6.28 mm. The left arm of the fenestrated segment had a diameter of 1.64 mm, and the right arm had a diameter of 2.25 mm. Lastly, the right anteroinferior cerebellar artery originated from the right arm of the BA fenestration (Figure 1).

The remaining cerebral arterial circle was typical.

## Discussion

The morphological variability of the ACA is estimated with a pooled prevalence of 6.25% [4]. The current literature describes only a few rare congenital variations of the ACA, one of which is the PPOA [4]. Previous imaging studies have reported the prevalence of this variant in 0.14% with magnetic resonance angiography (MRA) [3] and 0.26% with CTA [5]. Anatomical possibilities of the PPOA were previously described: type 1, PPOA termination as distal ACA; type 2, PPOA termination as the ethmoidal artery; type 3, PPOA termination in both distal ACA and PPOA; type 4, origin from an accessory MCA; and type 5, the absence of a hairpin turn [5,6]. In the current case, the PPOA observed could be classified as type 1.

Due to the anatomy of the PPOA, courses through the cribriform plate and a hairpin turn, some clinical implications were previously described. Aneurysms were identified on the variant artery that can be managed through the frontotemporal craniotomy or interhemispheric approach [7]. This pathological process can be justified due to the altered hemodynamics of the hairpin turn that represent an important risk factor for aneurysm formation [3,7]. Another important consideration is the presence of this variant during anterior skull base approaches. Surgeons should identify it preoperatively to prevent injury and bleeding. Both imaging modalities, CTA and MRA, can adequately illustrate the presence of PPOA [3,7].

The fenestration of the cerebral arterial circle has been a subject of extensive research. The proximal BA was considered the most common intracranial location of fenestration. Large retrospective studies have reported its prevalence that ranged from 1.10% (120/10927 patients) [8] to 2.07% (69/3327) [9]. Fenestrations have been correlated with altered hemodynamics and defects of the medial layer, resulting in turbulent flow at the VA-BA junction, therefore causing saccular aneurysms [8,9]. BA aneurysms have been treated with endovascular techniques rather than surgically [10]. However, in select cases where endovascular repair is not appropriate, the surgical approach has significant difficulties, especially in cases of fenestrations [11].

The current case describes the coexistence of PPOA and BA; thus, it is important to mention previous cases of concomitant variants. Previous cases described the presence of PPOA, along with an azygos pericallosal ACA [2], accessory MCA [7], and VA fenestration [3]. Moreover, BA fenestration was described in coexistence with ACA fenestration [12] and VA fenestration [13]. Therefore, to the authors’ knowledge, the concomitance of PPOA and BA fenestration was not identified in the current literature.

## Conclusions

In summary, this imaging report describes a unique and previously undocumented co-occurrence of a PPOA and a BA fenestration. The awareness of such variants is critical for clinicians involved in neurosurgical, endovascular, and skull base procedures, as these configurations may alter hemodynamics, predispose to aneurysm formation, and increase operative risk. Modern non-invasive imaging modalities, including CTA and MRA, remain the diagnostic tools of choice for identifying and characterizing these rare vascular anomalies.
